# Granzyme M has a critical role in providing innate immune protection in ulcerative colitis

**DOI:** 10.1038/cddis.2016.215

**Published:** 2016-07-21

**Authors:** F Souza-Fonseca-Guimaraes, Y Krasnova, T Putoczki, K Miles, K P MacDonald, L Town, W Shi, G C Gobe, L McDade, L A Mielke, H Tye, S L Masters, G T Belz, N D Huntington, G Radford-Smith, M J Smyth

**Affiliations:** 1 Immunology in Cancer and Infection Laboratory, QIMR Berghofer Medical Research Institute, Herston, Queensland 4006, Australia; 2 Faculty of Medicine, Dentistry and Health Sciences, University of Melbourne, Melbourne, Victoria 3010, Australia; 3 Division of Molecular Immunology, The Walter and Eliza Hall Institute of Medical Research, Parkville, Victoria 3052, Australia; 4 School of Medicine, University of Queensland, St Lucia, Queensland 4006, Australia; 5 Inflammation Division, The Walter and Eliza Hall Institute of Medical Research, Parkville, Victoria 3052, Australia; 6 Antigen Presentation and Immunoregulation Laboratory, QIMR Berghofer Medical Research Institute, Herston, Queensland 4006, Australia; 7 Signal Transduction Laboratory, QIMR Berghofer Medical Research Institute, Herston, Queensland 4006, Australia; 8 Centre for Kidney Disease Research, School of Medicine, University of Queensland at Translational Research Institute, St Lucia, Queensland 4006, Australia; 9 Inflammatory Bowel Disease Laboratory, QIMR Berghofer Medical Research Institute, Herston, Queensland 4006, Australia; 10 Department of Gastroenterology, Royal Brisbane and Women's Hospital, Herston, Queensland 4006, Australia

## Abstract

Inflammatory bowel disease (IBD) is an immunoregulatory disorder, associated with a chronic and inappropriate mucosal immune response to commensal bacteria, underlying disease states such as ulcerative colitis (UC) and Crohn's disease (CD) in humans. Granzyme M (GrzM) is a serine protease expressed by cytotoxic lymphocytes, in particular natural killer (NK) cells. Granzymes are thought to be involved in triggering cell death in eukaryotic target cells; however, some evidence supports their role in inflammation. The role of GrzM in the innate immune response to mucosal inflammation has never been examined. Here, we discover that patients with UC, unlike patients with CD, display high levels of GrzM mRNA expression in the inflamed colon. By taking advantage of well-established models of experimental UC, we revealed that GrzM-deficient mice have greater levels of inflammatory indicators during dextran sulfate sodium (DSS)-induced IBD, including increased weight loss, greater colon length reduction and more severe intestinal histopathology. The absence of GrzM expression also had effects on gut permeability, tissue cytokine/chemokine dynamics, and neutrophil infiltration during disease. These findings demonstrate, for the first time, that GrzM has a critical role during early stages of inflammation in UC, and that in its absence colonic inflammation is enhanced.

Inflammatory bowel disease (IBD) is a gut-associated inflammatory disorder, which stems from a dysfunctional mucosal immune response to commensal bacteria.^1^ As a multifactorial disease, IBD is the consequence of a complex interplay between environmental triggers, genetic susceptibility, and immunoregulatory defects, resulting in a pathogenesis that is still poorly understood.^2^ These interactions result in the inability of an individual to control the normal inflammatory response to pathogens in the gut, leading to a chronic state of sustained and inappropriate inflammation. IBD underlies disease states such as ulcerative colitis (UC) and Crohn's disease (CD), with symptoms including weight loss, abdominal pain, diarrhea, and rectal bleeding which often require intensive medical therapy and resective surgery.^3^ The pathogenesis of IBD, characterized by a defective mucosal immune response to microbial exposure in the gastrointestinal tract, is thought to be caused by a dysfunctional immune response to host microbiota, infection by specific pathogens, and/or a defective mucosal barrier to luminal pathogens.^1, 2^ IBD patients also have a high risk of developing colitis-associated colon cancer (CAC).^4^ Additionally, histological assessment of inflamed ileal and colonic segments from IBD patients typically shows increased infiltration of immune cells, particularly neutrophils, as well as crypt abscesses, mucin depletion, and ulcers—all correlating with the severity of small bowel and colonic tissue damage.^5^

Cytotoxic pathways mediated by lymphocytes directly trigger cell death in target cells.^6^ These cytotoxic pathways are mediated by proteins such as perforin, which mediates pore formation in the target cell surface and allows granzyme (Grz)s to enter the intracellular compartment and induce cell death.^7^ To date, five different Grzs have been identified in humans (GrzA, GrzB, GrzH, GrzK, and GrzM), whereas mice express eleven Grzs (GrzA, GrzB, GrzC, GrzD, GrzE, GrzF, GrzG, GrzK, GrzL, GrzM, and GrzN).^8, 9^ Walch *et al.*^10^ recently demonstrated that Grzs (GrzA and GrzB) directly kill bacteria through granulysin-mediated delivery, suggesting that Grzs act as microbial modulating factors. Moreover, recently GrzA was shown to be increased in the colon biopsies of UC patients undergoing treatment with Etrolizumab, a monoclonal antibody targeting the *β*7 integrin subunit. Higher levels of GrzA could predict which patients were more likely to benefit from the therapy; however, the precise mechanism of action of GrzA in UC remains to be addressed.^11^ GrzM was initially described as being constitutively expressed by natural killer (NK) cells,^12, 13^ and specifically associated with inflammation.^14^ This enzyme has been shown to preferentially cleave methionine and leucine residues in target cells, mediating direct, non-specific cell death.^15, 16^ More recently, GrzM was also shown to be an important mediator for the release of MIP-1*α* from NK cells, inducing NK cell and neutrophil recruitment during early microbial infection.^17^ We now observe that GrzM expression is increased in inflamed colon tissue samples from UC, but not CD patients. Further, GrzM-deficient (GrzM^−/−^) mice are more sensitive to a mouse model of IBD and IBD-induced colorectal cancer (CRC). These findings demonstrate, for the first time, that GrzM has a critical role in mediating the early stages of the gut mucosal immune response.

## Results

### GrzM expression is increased in the inflamed rectal tissues from UC patients

mRNA analysis of mucosal tissue biopsies from UC patients has been identified as a potential tool to investigate gene expression at different time points in this disease.^11^ To investigate whether GrzM may either be present or highly expressed in the inflamed intestinal segments of IBD patients, biopsies from inflamed and non-inflamed regions of the cecum, transverse colon, sigmoid colon, and rectum of UC patients; and from inflamed and non-inflamed regions of the ileum of CD patients were compared with tissue biopsies matched for intestinal segments from healthy controls. Biopsies were obtained at the time of colonoscopy, processed for tissue mRNA extraction and analyzed for GrzM mRNA (Table 1). Notably, GrzM expression was elevated specifically in the inflamed tissues of the rectal portions of UC patients, while no difference was detected in CD patients.

### GrzM is critical for colonic integrity during experimental dextran sulfate sodium-induced colitis

**Figure 1:**
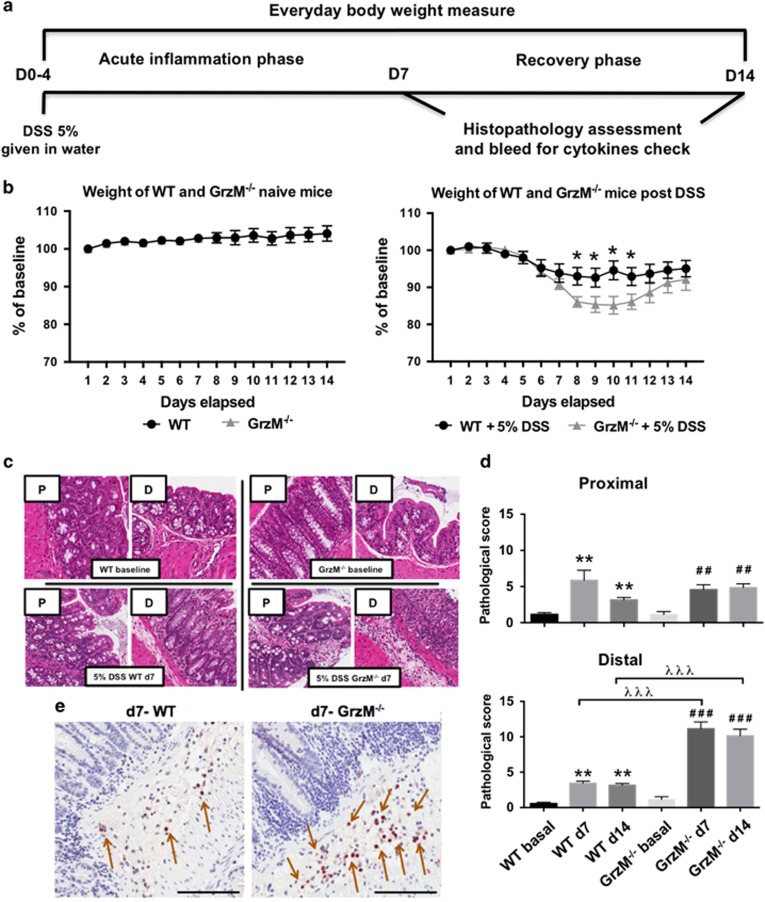
DSS-induced colitis reveals GrzM as a critical factor for disease protection. Mice were challenged with 5% DSS in the drinking water for 4 days, and samples were assessed 7 or 14 days post first DSS treatment day, according to the experimental design proposed in (**a**). (**b**) DSS-induced weight loss was measured daily throughout the 14-day experimental period. Statistical analysis was performed using Mann–Whitney test. Results are representative from pool of two experiments, and are expressed in mean±S.D. *n*=5 mice per experiment (*n*=10 total), and *P<0.05 was considered for statistical significance. (**c**) Representative histological sections (HE stain) from the proximal (P) and distal (D) colon parts from WT or GrzM-deficient mice at baseline, 7 days post 5% DSS (d7), and 14 days post 5% DSS (d14). (**d**) Histopathological scoring quantification from colons post 5% DSS. Statistical analysis was performed using Mann–Whitney test. Results are expressed in mean±S.E.M. *n*=6 mice per group, *P<0.05, ** (or ##) *P*<0.01, and *** (or ### or λλλ) *P*<0.001. *Comparison between WT group, ^#^comparison between GrzM-deficient group, and ^λ^comparison between WT and GrzM-deficient groups. (**e**) Ly6G immunohistochemistry illustrates the increased neutrophil (Ly6G^+^ revealed by DAB stain (brown die, indicated by brown arrows) in representative areas of the colonic lamina propria of WT and GrzM-deficient mice post 7 days after DSS challenge

Following on from our finding that GrzM expression was elevated in the rectal tissues of UC patients, we set out to investigate whether GrzM expression was favorable or unfavorable in the UC disease course. By taking advantage of the GrzM-deficient mice previously described by our group,^18^ we performed a screen with dextran sulfate sodium (DSS)-induced colitis (Figure 1a), a mouse experimental model of human UC.^19^ We observed that GrzM^−/−^ mice were susceptible to weight loss following a 5% DSS dose (Figure 1b). In concert, by using a well-established histopathology characterization and scoring system,^20^ we observed that the central-distal portion of the colon of GrzM^−/−^ mice post DSS treatment was dramatically affected (Figures 1c and d). In agreement with the increased gut pathology, enhanced neutrophil recruitment was detected in the lamina propria region post DSS treatment (Figure 1e). To investigate whether the enhanced colonic disease phenotype seen in GrzM-deficient mice would also be observed in other experimental models that are dependent on gut integrity, we performed experiments using *Toxoplasma gondii*^21^ and *Citrobacter rodendium*;^22^ however, we failed to detect any difference between WT and GrzM^−/−^ mice in these conditions (data not shown).

### GrzM expression inhibits excessive neutrophil infiltration and promotes gut integrity

**Figure 2:**
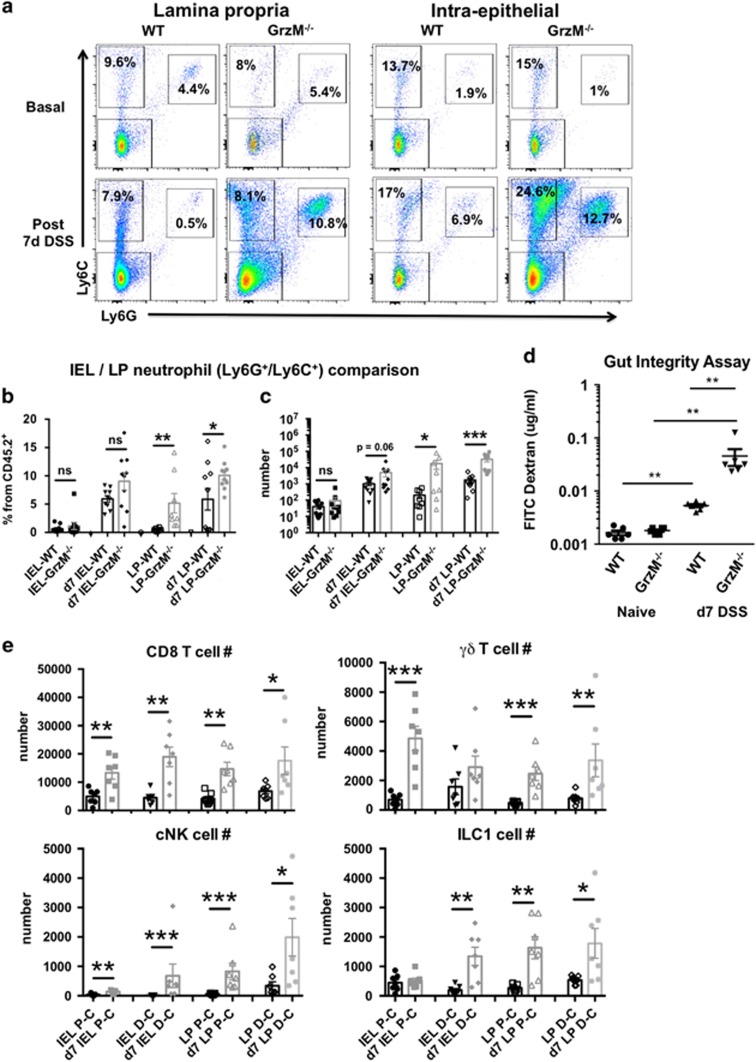
DSS treatment induced higher neutrophil infiltration in colons of GrzM-deficient mice. (**a**) Representative dot blots from both colon lamina propria and intraepithelial phase of WT and GrzM-deficient mice pre and post 7 days of (7d) 5% DSS treatment. Neutrophils were gated from leukocyte gate (CD45.2^+^) as Ly6C^+^Ly6G^+^. (**b**) Neutrophils were compared within their respective % of CD45.2+ leukocytes from both lamina propria (LP) and intraepithelial (IEL) parts. (**c**) Bead-based absolute counting was utilized to quantify neutrophils from both lamina propria and epithelium. Results are expressed in mean±S.E.M., and *P<0.05, **P<0.01, and ***P<0.001 were considered as statistically significant by Mann–Whitney test. Results are representative from the pool of two independent experiments with *n*=5 in each group (*n*=10, total). (**d**) Gut integrity of naive WT and GrzM-deficient animals, or post 7 days after DSS challenge, was assessed by measuring the *in vivo* gut permeability post oral gavage administration of FITC-dextran for 4 h and then respective FITC detection in plasma. Results are expressed in mean±S.E.M., and **P<0.01 was considered as statistically significant by Mann–Whitney test. Results are representative from the pool of two experiments with *n*=3 in each group (*n*=6, total). (**e**) CD8 T cell, *γδ* T cell, conventional NK (cNK), and ILC1 cell quantification from the proximal-central (PC) and distal-central (DC) parts of IEL and LP colonic fractions, post 7 days after DSS challenge in WT mice. Results are expressed in mean±S.E.M., and *P<0.05, **P<0.01, or ***P<0.001 was considered as statistically significant by Mann–Whitney test. Results are representative from *n*=7 independent biological replicates for each group

Neutrophils are critical for bacterial leakage control and maintenance of homeostasis; however, excessive recruitment and activation of these cells may trigger mucosal injury and consequently worsen disease symptoms.^23^ To verify whether GrzM deletion increased the colonic infiltration by neutrophils at the steady state or during DSS-induced colitis, homogenates of the intraepithelial portion of the colon and lamina propria were assessed. In concert, GrzM-deficient mice displayed increased neutrophil infiltration, especially in lamina propria fractions after DSS challenge (Figures 2a–c). As a consequence of excessive neutrophil activation, gut integrity can be damaged during IBD, and can allow luminal antigen and bacterial leakage into the subepithelial tissues, resulting in enhanced inflammation.^24^ To investigate whether gut integrity is affected in the steady state or whether the damage is increased following DSS treatment, we assessed intestinal permeability using an FITC-dextran absorption assay.^25^ Gut integrity was preserved in GrzM^−/−^ mice during the steady state; however, a dramatically elevated translocation of FITC-dextran from gut to the circulation was detected when these mice were challenged with DSS, suggesting that the presence of GrzM is critical for gut integrity (Figure 2d). The gut integrity dysfunction followed by induction of inflammation triggers increased neutrophil chemotaxis and infiltration into the inflamed tissue.

In addition, to investigate whether colonic lamina propria or intraepithelial cells, specifically in the proximal or distal fractions, were also infiltrated by lymphocyte subsets (previously described as potential expressers of GrzM^26^), we also assessed the infiltration of CD8 T cells, *γδ* T cells, or Group 1 innate lymphoid cells (which incorporate NK cells and ILC1s^27^) post DSS in WT mice. The gating strategy for the quantification of these cell subsets from colonic tissue is shown in Supplementary Figure 1. In agreement with the increased neutrophil infiltration, all these lymphocyte subsets were increased in numbers in the lamina propria in both distal and proximal parts (Figure 2e). In the intraepithelial fraction, predominant CD8 T cells and NK cells were increased in the intraepithelial fractions in both distal and proximal parts, while *γδ* T cells were in higher levels in the proximal part and ILC1s increased in the distal part. Although all the potential GrzM-expressing lymphocyte subsets were increased in the colonic tissue post DSS challenge, the specific deficiency or depletion of CD8 T cells, *γδ* T cells, or NK/ILC1 cells did not mimic the GrzM-deficient mice phenotype (Supplementary Figure 2A–D). Perforin (Prf1) is a molecule expressed by cytotoxic lymphocytes that are used in killing by transiently inducing pore formation target cells and allowing Grzs to be internalized and induce cell death.^28^ Although perforin can act with Grzs to induce cytotoxic events, the effects of GrzM in UC appeared to be perforin independent, since the Prf1-deficient mice displayed no enhancement of colitis post DSS challenge (Supplementary Figure 2E). Colon length is a quantitative measure of disease severity where the extent of inflammation directly correlates to increased edema, leading to an overall shortening of the colon.^29^ In concert, elevation of pro-inflammatory cytokines also correlates with increased disease pathology in IBD.^30^ Although we observed enhanced colon length shrinkage in GrzM- and TCR*δ*-deficient mice after DSS-induced UC, in agreement with the weight loss data we only observed elevation of G-CSF and TNF in the serum of GrzM-deficient mice (Supplementary Figure 2F–H). Our results indicate that the GrzM-mediated colonic protection involves a mechanism that might require a complex participation of multiple factors to promote protection in UC.

### GrzM expression protects colonic epithelium from inflammation-induced CRC

**Figure 3:**
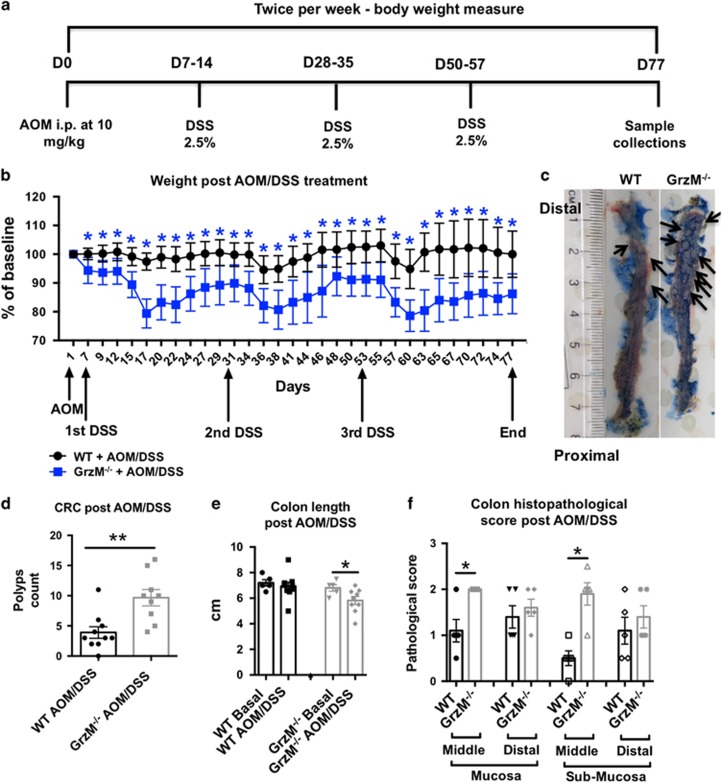
Enhanced inflammation-induced CRC is observed in GrzM-deficient mice. Mice were challenged with AOM/DSS, and samples were assessed according to the experimental design proposed in (**a**). (**b**) DSS-induced weight loss was measured twice/week throughout the experimental period of 77 days. Statistical analysis was performed using multiple *T* tests using the Sidak–Bonferroni method. Results are expressed in mean±S.D. *n*=15 mice per group, and *P<0.05 was considered for statistical significance. (**c**) Macroscopical representative images are displayed for one representative WT and one GrzM-deficient mice at the end of the experiment. A cotton stick containing Alcian Blue solution (1%) was used to swap along the opened colon tissue to enhance the CRC polyp visualization, indicated by the black arrows. (**d**) Colorectal (CRC) polyp numbers were counted at the end point of the experiment. Statistical analysis was performed using Mann–Whitney test. Results are expressed in mean±S.E.M., *n*=10 per group, and **P<0.01 was considered for statistical significance. (**e**) Colon length at the end point of the experiment is represented in cm. Statistical analysis was performed using Mann–Whitney test. Results are expressed in mean±S.E.M., *n*=5–10 mice per group, and *P<0.05 was considered for statistical significance. (**f**) Colon histopathology at the end point of the experiment is represented in pathological score from the mucosa and submucosa tissue sections. Statistical analysis was performed using Mann–Whitney test. Results are expressed in mean±S.E.M., *n*=5 mice per group, and *P<0.05 was considered for statistical significance

We next assessed whether GrzM^−/−^ mice would also be more susceptible to colon cancer development. Colonic epithelial oncogenesis can either be developed by sporadic progression due to genetic predisposition or following an inflammation-associated trigger.^31^ Azoxymethane (AOM) is a highly mutagenic chemical compound that causes K-ras mutations. When used repeatedly via intraperitoneal administration, it can trigger sporadic CRC, and when used before DSS-induced inflammation it can trigger inflammation-associated CRC.^32^ We failed to observe any phenotype of sporadic CRC predisposition in GrzM^−/−^ mice in response to repetitive AOM treatment (data not shown). However, when the inflammation-induced CRC model was examined (Figure 3a), the GrzM^−/−^ mice clearly displayed susceptibility to the AOM-DSS-induced CRC demonstrated by enhanced weight loss cycles (Figure 3b). As expected, GrzM-deficient mice also displayed an increased number of macroscopically visible colorectal polyps (Figures 3c and d). Colon length was another indicator of increased inflammation, since we observed a significant shortening of colon length in GrzM-deficient mice compared with the wild-type (WT) control mice at the same stages of inflammation (Figure 3e). This suggests that the severity of inflammation during the AOM-DSS model was enhanced in GrzM-deficient mice. This was corroborated by the measurement of an enhanced histopathology scoring of the middle parts of both mucosa and submucosa sections in the colon of GrzM-deficient mice at the end point of the experiment (Figure 3f). We also observed that the effects of AOM-DSS in GrzM-deficient mice had a systemic impact, since the mice displayed increased mesenteric lymph nodes and spleen sizes, as well as increased leukocyte numbers in both compartments (data not shown).

Regarding the potential GrzM expression by colonic epithelial cells, Wang *et al.*^33^ recently showed mouse GrzM detection in mouse colon cancer cells using the anti-mouse GrzM antibody clone aa31-257. In addition to this, another anti-mouse GrzM antibody clone P-15 also recently became commercially available. To investigate whether these available tools could help us decipher in which exact colon cellular compartment GrzM is expressed, we initially assessed whether the staining of both aa31-257 and P-15 antibody clones was specific to mouse GrzM. We performed tissue protein extraction from the colons of WT and GrzM-deficient mice and subsequently performed western blot detection according to the manufacturer's instructions. However, we found that both aa31-257 and P-15 clones were highly non-specific, as there was staining in all the GrzM^−/−^ samples (Supplementary Figure 3). Among immune cells, NK cells have been described as the major expressers of GrzM, while expression in other innate T cells (CD8, and *γδ* T) can occur at a reduced level.^26, 34^ To test whether any of these cells could demonstrate an increased mean fluorescence intensity when labeled with the aa31-257 antibody, intracellular staining and flow-cytometry analysis were performed. However, the aa31-257 staining was again revealed as highly non-specific since all GrzM^−/−^ cells stained positive (Supplementary Figure 4). Given that the mouse anti-human GrzM mAb does not cross-react with mouse GrzM,^35^ these results suggest that there are still no available tools to adequately detect mouse GrzM.

### GrzM deficiency results in a distinct cytokine/chemokine profile in the colon during experimental colitis

Several cytokines and chemokines are involved in the ‘physiological inflammation' of the normal intestine, and may also have important roles in the pathogenesis of IBD orchestrated by the dynamics of the inflammatory response.^30^ We performed a kinetic analysis to determine which cytokines/chemokines, from a total of 23 targets, would be affected during DSS-induced colitis progression in the distal colonic segment of GrzM-deficient mice (Experimental summary in Table 2). Strikingly, G-CSF, IL-1*α*, IL-1*β*, IL-17A, MIP1*α*, MIP1*β*, RANTES, and TNF*α* levels were elevated in GrzM-deficient mice during disease progression, especially at the end point of the experiment (after 7 days of DSS treatment) (Supplementary Figure 5). In contrast, IFN-*γ*, IP-10, and IL-23 were decreased in GrzM-deficient mice during the colitis progression. (Supplementary Figure 6). We failed to detect any GM-CSF, IL-2, and IL-13 cytokines in the colon homogenates (data not shown), while KC, MCP-1, IL-6, IL-10, IL-12p70, IL-21, IL-22, IL-28A/B, and TGF-*β*1 levels were unaffected (data not shown). Our results indicate that the presence of GrzM is critical for the appropriate cytokine/chemokine response during DSS-induced colitis.

## Discussion

Our current study demonstrates a relationship between GrzM and gut mucosal protection in UC. Grzs are serine proteases produced by cytotoxic lymphocytes that are well known for their potential to induce cell death in target eukaryotic cells. However, previous evidence has shown that GrzM may also participate in inflammatory processes, such as MIP-1*α* regulation during *Listeria monocytogenes* infection, and the regulation of secretion of pro-inflammatory cytokines during endotoxic shock.^14, 17^ Here, we have shown that GrzM also has a role during the disease progression of UC. Following up on our clinical evidence that GrzM expression is elevated exclusively in the inflamed rectum (distal part of the colon) of UC patients, we have taken advantage of the GrzM-deficient mice to demonstrate in a series of *in vivo* experiments that host GrzM expression significantly affects the regulation of colonic inflammation. To initially address whether host GrzM would provide a protective advantage or a disadvantage during experimental colitis, we elected the DSS-induced colitis model as a UC experimental model as it has a number of similarities with human UC.^19^ We observed that host GrzM was critical for the protection against colitis, and that its absence results in a significant worsening of colitis in mice following DSS treatment. Surprisingly, the most affected colonic areas in the GrzM-deficient mice were the distal-central segments, which correspond to the distal colon and rectum in UC patients, where we observed elevated GrzM expression during active colitis. These results strongly suggest that GrzM can act directly to induce colonic protection during UC in this specific anatomical region of the gut.

Certain host mutations were described as enhancers of gut permeability, resulting in a dysfunctional immune response to host microbiota, infection by specific pathogens, and/or a defective mucosal barrier to luminal pathogens.^1^ To assess whether GrzM-deficient mice naturally display gut permeability at steady state, or whether it is enhanced during DSS challenge, we performed a gut integrity assay based on FITC-dextran leakage from the gut to the blood compartment. We have identified that gut integrity was not compromised at steady state, but worsened during DSS treatment. Our results also reinforced the finding that enhanced colon damage in GrzM-deficient mice seemed to be limited to the experimental model used, as no such phenotype was observed in other gut-related infection/pathology models including *Toxoplasma gondii* and *Citrobacter rodentium* infections. During inflammatory tissue damage occurring in UC, inflamed tissues typically represent trans-epithelial migration of neutrophils, which can alter the gut barrier function by enhancing the epithelial paracellular permeability during disease progression.^36^ Along with the enhanced pathology and increased gut permeability seen during DSS treatment, as expected, we also observed an enhanced neutrophil infiltration in the colonic lamina propria in GrzM-deficient mice.

IBD-induced CRC is a subsequent clinical complication that can account for up to 15% of all deaths among IBD patients, and IBD patients are at least six times more likely to develop CRC. To explore whether host GrzM is involved in protection against CRC development, we assessed well-established models that mimic both IBD-induced CRC in GrzM-deficient mice and their respective WT controls.^32^ As expected, the absence of GrzM expression resulted in an enhanced AOM–DSS-induced CRC, which is exclusively dependent on enhanced colonic inflammation. Another study recently showed that GrzM expression in epithelial cells contributes to chemoresistance and epithelial–mesenchymal transition (EMT) during colon carcinogenesis, demonstrating that GrzM expression increases in colon cancer tissues and cell lines upon enhanced EMT phenotype.^33^ We could not assess EMT in our model, but when we tested the anti-mouse GrzM antibodies used in the study by Wang *et al.*,^33^ these reagents were highly non-specific as determined by negative control GrzM-deficient samples. Precise cellular GrzM expression assays remain controversial and limited, as there is still no efficient/specific method to detect GrzM in mice, and GrzM-reporter mice are still not available. A technique of tagging GrzM with a fluorescent label and then using live-cell imaging may demonstrate whether GrzM is secreted from NK cells or whether it has intracellular functions instead; however, a limiting factor is that recombinant GrzM is not commercially available.

Several cytokines and chemokines have an important role in the pathogenesis of IBD, and modulation of specific cytokines is a current clinical therapeutic strategy (e.g., neutralizing antibodies to TNF*α*).^30^ Considering that different colonic segments can display different colitis intensities according to our previous data, we homogenized in tissue protein extraction buffers and screened 23 cytokines/chemokines from the distal colon segments at different time points following DSS treatment. Certain pro-cytokines and chemokines were elevated at later time points following DSS treatment in the GrzM-deficient mice: G-CSF, IL-1*α*, IL-1*β*, IL-6, IL-17A, MIP-1*α*, MIP-1*β*, RANTES, and TNF*α*. IL-1 cytokines and TNF*α* are classically produced by macrophages or dendritic cells (DCs) via TLR sensing of the commensal microbiota.^37^ TNF*α* neutralization, but not IL-1 neutralization, was already shown to be effective in DSS-induced colitis by favoring mucosal healing.^38^ In addition, IL-1 and TNF*α* were also demonstrated to be associated with colorectal tumorigenesis by contributing to tissue inflammation-induced oncogenesis.^39, 40^ This evidence corroborates our findings of enhanced colitis and inflammation-induced tumorigenesis outcomes in GrzM-deficient mice subjected to DSS challenge. Chemokines are also critical factors in regulating immune cell trafficking to inflammatory sites. MIP-1*α*, MIP-1*β*, and RANTES are chemokines that have already previously been shown to contribute to the exacerbation of IBD in experimental mouse models via overzealous neutrophil attraction and infiltration of the colonic tissues.^41, 42, 43, 44^ In addition, G-CSF and IL-17A are important cytokines that activate neutrophil innate functions to control bacterial infection, but in excess, they have already been described as contributing to exaggerated neutrophil activation and consequent tissue damage during DSS-induced colitis.^45, 46^ This evidence can provide an explanation for our observations of enhanced neutrophil infiltration of the colonic tissues of GrzM-deficient mice after DSS treatment, and the associated tissue damage and enhanced disease outcome. IFN-*γ* and IFN-*γ*-induced protein (IP-10) were also observed in lower concentrations in the GrzM-deficient epithelium during d1 of colitis. The functions of IFN-*γ* and IP-10 are to promote Th1 differentiation and to attract these cells to the inflammation site, respectively.^47, 48^ Th1-related cells and cytokines were previously shown to represent a favorable prognostic sign in human CRC initiation.^49^ The decreased IFN-*γ* and IP-10 levels in distal colon homogenates might also suggest that the anticancer Th1 response may be reduced in GrzM-deficient mice. Further research is necessary to elucidate the specific stage of the disease progression in which GrzM has a role, and to demonstrate that this enzyme might offer complementary benefits for treating or preventing UC.

## Materials and Methods

### Clinical samples, RNA isolation, and sequencing

Patients with a confirmed diagnosis of either UC or CD undergoing colonoscopy for surveillance or assessment of their disease, and healthy controls undergoing screening colonoscopy because of a family history of CRC, were recruited into an established, ethically-approved study investigating the natural history and pathogenesis of IBD.

### Mice

C57BL/6J WT mice were purchased from the Walter and Eliza Hall Institute of Medical Research and housed at the QIMR Berghofer Medical Research Institute. C57BL/6 GrzM-deficient mice were previously described by our group,^18^ and were bred at the QIMR Berghofer Medical Research Institute. C57BL/6 perforin (Prf1)-deficient^50^ and TCR*δ*-deficient mice^51^ were bred at the QIMR Berghofer Medical Research Institute. Conditional transgenic NK cell-deficient mice (NKp46cre × Mcl-1^fl/fl^) were previously described by our group,^52^ and were bred at the Walter and Eliza Hall Institute of Medical Research. In certain experiments, anti-CD8 (clone 53.5.8), or respectively control IgG (control group), was administered in a 100 *μ*g i.p. dose at day −1, day 0, and day 7 relatively to DSS administration to induce CD8 T-cell depletion.^53^ All mice used were females between the ages of 8 and 14 weeks. All experiments were approved by QIMR Berghofer Medical Research Institute and Walter and Eliza Hall Institute of Medical Research animal ethics committees.

### *In vivo* DSS-induced IBD

To induce colitis, WT and other indicated genotypes/antibody-depletion group were treated with either 0 or 5% DSS (molecular mass 40–50 kDa; USB, Affymetrix Inc, Ohio, USA), dissolved in their sterile drinking water and provided *ad libitum* over a 4-day period to simulate the inflammatory conditions experienced during IBD, according to a previously described protocol.^29^ A baseline weight was recorded for each mouse before treatment and weight was then monitored daily for the subsequent 2-week period. After 4 days, the DSS treatment was replaced with normal autoclaved water to allow for inflammation progression and resolution. On days 7 (acute inflammation) and 14 (tissue recovery period) post DSS treatment, mice were killed via CO_2_ asphyxiation, and biopsies were taken to investigate the inflammatory conditions.

### *In vivo* AOM-DSS-induced CRC

AOM/DSS-induced CRC was performed as previously described.^32^ Briefly, WT and GrzM-deficient mice were individually tagged, had their body weights measured, and were injected i.p. with 10 mg/kg of AOM (Sigma-Aldrich, St. Louis, MO, USA) diluted in PBS. After 7 days post AOM treatment, 2.5% DSS was administered in their drinking water to simulate the chronic inflammatory conditions experienced prior the development of CRC polyps. After 7 days, the DSS treatment was replaced by normal autoclaved water to allow for inflammation progression and resolution during the following 2 weeks. Another two cycles of 2.5% DSS treatment for 7 days and normal drinking water for 14 days were performed. During the treatment period, weight was monitored twice a week. After 77 days post AOM injection (tissue recovery period), mice were killed via CO_2_ asphyxiation, and biopsies were taken to investigate the inflammatory conditions.

### Histology and histopathology assessment

Colonic tissues from unchallenged, DSS, or AOM-DSS treated mice were rolled into Swiss rolls and fixed in PBS–Formalin 10% for 48 h and then submitted for paraffin processing and HE or GRAM staining performed routinely by the QIMR Berghofer Medical Institute's Histology Facility. Slides were then scanned using an Aperio XT slide scanner using × 40 scan magnification. Histopathology in colon samples from DSS-induced UC experiments was assessed as previously described,^20^ using the following parameters for histopathological scoring: Crypt integrity: 0=normal; 1=irregular crypts, including the presence of apoptosis; 2=mild crypt loss, and/or mild epithelial loss from surface of bowel; 3=severe crypt loss; 4=complete crypt loss with an intact epithelial cell layer; 5=complete loss of crypts and surface epithelium (<crypt width); and 6=complete loss of crypts and surface epithelium (>crypt width). Infiltration of muscle: 0=normal; 1=mild; 2=moderate; and 3=severe. Infiltration of inflammatory cells into mucosa: 0=normal; 1=mild; 2=moderate; and 3=severe. Infiltration of inflammatory cells into submucosa: 0=normal; 1=mild; 2=moderate; and 3=severe. Scores were added, resulting in a total scoring range of 1–17. Histopathology in colon samples from AOM-DSS-induced CRC experiments was assessed as previously described by our group,^54^ using the following parameters for histopathological scoring: Epithelial integrity: 0=normal; 1=hyper proliferation; 2=<50% crypt loss; 3=>50% crypt loss; 4=complete crypt loss; and 5=ulcer. Presence of inflammatory cells: 0=none; 1=mild; 2=moderate; and 3=severe. Scores were added, resulting in a total scoring range of 0–8.

### Sample preparation for flow-cytometry analysis

Mesenteric lymph nodes and spleens were homogenized in PBS into 40 *μ*m cell strainers, followed by red blood cell (RBC) lysis. Colonic lamina propria and intraepithelial leukocytes (IELs) were prepared as previously described.^22^ Fresh colons were harvested from CO_2_ killed mice, cleaned by flushing 10 ml of cold PBS using a gavage syringe needle, cut into ~0.5 cm sections and dissociated in 2% FCS HANKS Ca^+^ Mg^+^-free media with 5 mM EDTA for 30 min at 37 °C with gentle rotation to obtain the IEL fraction (supernatant). The subsequent tissues were then digested for 45 min in 2% FCS RPMI 2 mg/ml Collagenase III (Worthington Biochemical, Lakewood, NJ, USA), 0.4 U Dispase (Invitrogen, Thermo Fisher Scientific, Grand Island, NY, USA), and DNAse 1 *μ*g/ml (Roche Diagnostics USA, Indianapolis, IN, USA) for isolation of lamina propria leukocytes. Cells from both IEL and lamina propria fractions were further purified by centrifugation on 40%/80% Percoll gradient for 20 min at 900 × *g* without breaks. Cells harvested from single-cell suspensions from various organs were incubated for 15 min in Fc blocking buffer (2.4G2 antibody). Cells were then stained with the following antibodies: anti-mouse -B220 (RA3-6B2), -CD3 (17A2), -CD4 (GK1.5), -CD8 (53-6.7) -CD19 (1D3), -Foxp3 (FJK-16S) -*γδ*TCR (GL3), -Ly6C (HK1.4), -Ly6G (1A8), -NKp46 (29A1.4), -NK1.1 (PK136), and -TCRβ (H57-597). All mAbs were purchased from eBiosciences (San Diego, CA, USA), BD Biosciences (San Jose, CA, USA), or Biolegend (San Diego, CA, USA). Zombie Yellow, or Zombie UV, Fixable Viability Kit (Biolegend) was used to assess viability. Acquisition was performed using LSR II Fortessa Flow Cytometer (BD Biosciences). Analysis was achieved using Flowjo (Tree Star, Ashland, OR, USA) software.

### Colon homogenates and cytokine detection

Fresh colons biopsies from unchallenged (d0) or 5% DSS-treated mice (d1, d3, d5, and d7) were flushed with 10 ml of cold PBS using a gavage syringe needle for stool content cleaning. Colons were then gently dried with absorbent tissue, length was measured with support of a ruler to allow the separation of distal colon parts, which were then weighed and homogenized as previously described^55^ using T-PER Tissue Protein Extraction Reagent (Thermo Fisher Scientific Life Sciences, Waltham, MA, USA; supplemented with PhosSTOP Phosphatase and complete Mini protease inhibitor tablets (Roche)), according to the manufacturer's instructions. All samples were stored at −80 °C until analysis. After filtering samples in 40 *μ*m filters, and centrifugation at 10 000 × *g* to pellet debris, the concentrations of a panel of cytokines and chemokines in supernatants were measured as previously described.^56^ The detection methods applied were either by using Cytometric Bead Array (CBA) technology (BD Biosciences) according to the manufacturer's instructions (for GM-CSF, G-CSF, IL-1*α*, IL-1*β*, IL-2, IL-6, IL-10, IL-13, IL-17A, KC, MCP-1, MIP-1*α*, MIP-1*β*, RANTES, TNF*α*) or by using ELISA Duoset Kits (R&D Systems, Minneapolis, MN, USA) according to the manufacturer's instructions (for IFN-*γ*, IL-12p70, IL-21, IL-22, IL-23, IL-28A/B, TGF-*β*1). In all cases, cytokine levels were normalized to the weight of the respective colon explant.

### Gut integrity assay

Gut integrity was assessed before or after 4 days of treatment with 5% DSS as previously described.^25^ Naive or DSS-treated mice (d7 post DSS) were kept without food or water for 4 h, and treated by oral gavage with 300 *μ*l of FITC-dextran molecular weight 4000 (Sigma-Aldrich) at 60 mg/ml. Plasma was obtained after 4 h of FITC-dextran treatment, diluted 1 : 1 with PBS and fluorescence was read in a plate reader (485/535 nm) using a standard curve.

### Statistical analysis

Statistical analysis was achieved using Graph Pad Prism (La Jolla, CA, USA) or SPSS Software (Chicago, IL, USA). Data were considered to be statistically significant where the *P-*value was equal to or less than 0.05. Statistical tests used were the Mann–Whitney test or one-way ANOVA with Tukey's *post hoc* test for treatments/genotypes comparison, multiple T tests using the Sidak–Bonferroni method for weight loss comparison, and the Mantel–Cox Log Rank test for survival.

## Figures and Tables

**Table 1:** High levels of GrzM expression found exclusively in inflamed rectum portions of UC patients

							**95% CI**	
	***N***	**Mean**	**S.D.**	**S.E.**	**Lower bound**	**Upper bound**	***P*-value**	***P*.adjust**
*Transverse colon in UC*								
C	22	6.47	0.40	0.09	6.29	6.65		
UC.I	15	6.43	0.30	0.08	6.27	6.60	0.048	0.338
UC.NI	17	6.21	0.25	0.06	6.08	6.34		
Total	54	6.38	0.35	0.05	6.28	6.47		
*Sigmoid in UC*								
C	22	6.32	0.29	0.06	6.19	6.45		
UC.I	32	6.42	0.32	0.06	6.31	6.53	0.335	0.361
UC.NI	14	6.31	0.21	0.06	6.19	6.43		
Total	68	6.36	0.29	0.03	6.29	6.43		
*Rectum in UC*								
C	22	6.29	0.18	0.04	6.21	6.37		
**UC.I**	**29**	**6.51**	**0.41**	**0.08**	**6.35**	**6.67**	0.018*	0.028*
UC.NI	11	6.26	0.13	0.04	6.18	6.35		
Total	62	6.39	0.32	0.04	6.31	6.47		
*Cecum in UC*								
C	22	6.38	0.29	0.06	6.25	6.50		
UC.I	10	6.28	0.16	0.05	6.16	6.39	0.201	0.403
UC.NI	16	6.25	0.13	0.03	6.18	6.32		
Total	48	6.31	0.23	0.03	6.25	6.38		
*Ileum in CD (non-inflamed)*								
C	21	6.609	0.424	0.093	6.416	6.802		
CD.NI	21	6.572	0.386	0.084	6.396	6.747	0.766	0.825
Total	42	6.590	0.401	0.062	6.465	6.715		
*Ileum in CD (inflamed)*								
C	21	6.609	0.424	0.093	6.416	6.802		
CD.I	25	6.429	0.390	0.078	6.268	6.590	0.140	0.656
Total	46	6.511	0.411	0.061	6.389	6.633		

Samples from inflamed (I) or non-inflamed (NI) areas from different anatomical parts of the colons of ulcerative colitis (UC: transverse, sigmoid, rectum, and cecum) or Crohn's disease (CD: Ileum) patients were screened and analyzed for GrzM mRNA expression. Statistical analysis was performed using one-way ANOVA, where *P<0.05 was considered for statistical significance.

**Table 2:** Temporal cytokine/chemokine profiles in distal colon of GrzM-deficient mice during experimental DSS-induced UC

	**D0**	**D1**	**D3**	**D5**	**D7**
GM-CSF	nd	nd	nd	nd	nd
G-CSF	nd	nd	nd	ns	↑↑↑
IFN-*γ*	ns	↓↓↓	ns	ns	ns
IL-1*α*	nd	nd	nd	ns	↑↑↑
IL-1*β*	nd	nd	nd	ns	↑↑↑
IL-2	nd	nd	nd	nd	nd
IL-6	nd	nd	ns	ns	ns
IL-10	nd	nd	nd	nd	ns
IL-12p70	ns	ns	ns	ns	ns
IL-13	nd	nd	nd	nd	nd
IL-17A	nd	nd	nd	↑	nd
IL-21	ns	ns	ns	ns	ns
IL-22	ns	ns	ns	ns	ns
IL-23	ns	ns	↓↓	ns	ns
IL-28A/B	ns	ns	ns	ns	ns
IP-10	ns	↓	ns	ns	ns
KC	nd	nd	nd	ns	ns
MCP-1	nd	nd	nd	nd	ns
MIP-1α	nd	nd	nd	nd	↑↑↑
MIP-1β	nd	nd	nd	ns	↑↑↑
RANTES	ns	ns	ns	↑	ns
TGF-β1	ns	ns	ns	ns	ns
TNFα	nd	nd	nd	↑	ns

Abbreviations: nd, not detected; ns, not significant. Distal part of colons of WT or GrzM-deficient mice was harvested in different time points after 5% DSS challenge (days 0, 1, 3, 5, and 7), homogenized in tissue protein extraction buffer, and analyzed for the indicated cytokines/chemokines. Pool of two independent experiments of *n*=5 mice each (total *n*=10). Statistical analysis was performed using one-way ANOVA followed by Tukey's *post hoc* test, where *P<0.05 (↑ for significantly higher, or ↓ for significantly lower than WT), **P<0.01 (↑↑ for significantly higher, or ↓↓ for significantly lower than WT), and ***P<0.001 (↑↑↑ for significantly higher, or ↓↓↓ for significantly lower than WT) were considered for statistical significance.

• Pro-inflammatory neutrophil-related cytokines/chemokines analyzed: GM-CSF, G-CSF, IL-17A, and KC.

• Chemokines analyzed: IP-10, MCP-1, MIP-1*α*, MIP-1*β*, and RANTES.

• Pro-inflammatory cytokines analyzed: IFN-*γ*, IL-1*α*, IL-1*β*, IL-2, IL-6, IL-12, IL-13, IL-21, IL-23, IL-28A/B, and TNF*α*.

• Anti-inflammatory, tissue repair proteins: IL-10, IL-22, and TGF-*β*1.

## Abbreviations

AOM: azoxymethane
CD: Crohn's disease
CAC: colitis-associated colon cancer
CRC: colorectal cancer
DSS: dextran sulfate sodium
EMT: epithelial–mesenchymal transition
Grz: granzyme
IBD: inflammatory bowel disease
IEL: intraepithelial leukocyte
ILC: innate lymphoid cell
LP: lamina propria
NK: natural killer
Prf1: perforin
RBC: red blood cell
UC: ulcerative colitis
WT: wild type
