# Supplementary figures and images for: Granzyme M has a critical role in providing innate immune protection in ulcerative colitis
Source: Cell Death Dis. 2016 Jul 21;7(7):e2302–. doi: 10.1038/cddis.2016.215 (PMC4973354; doi:10.1038/cddis.2016.215)

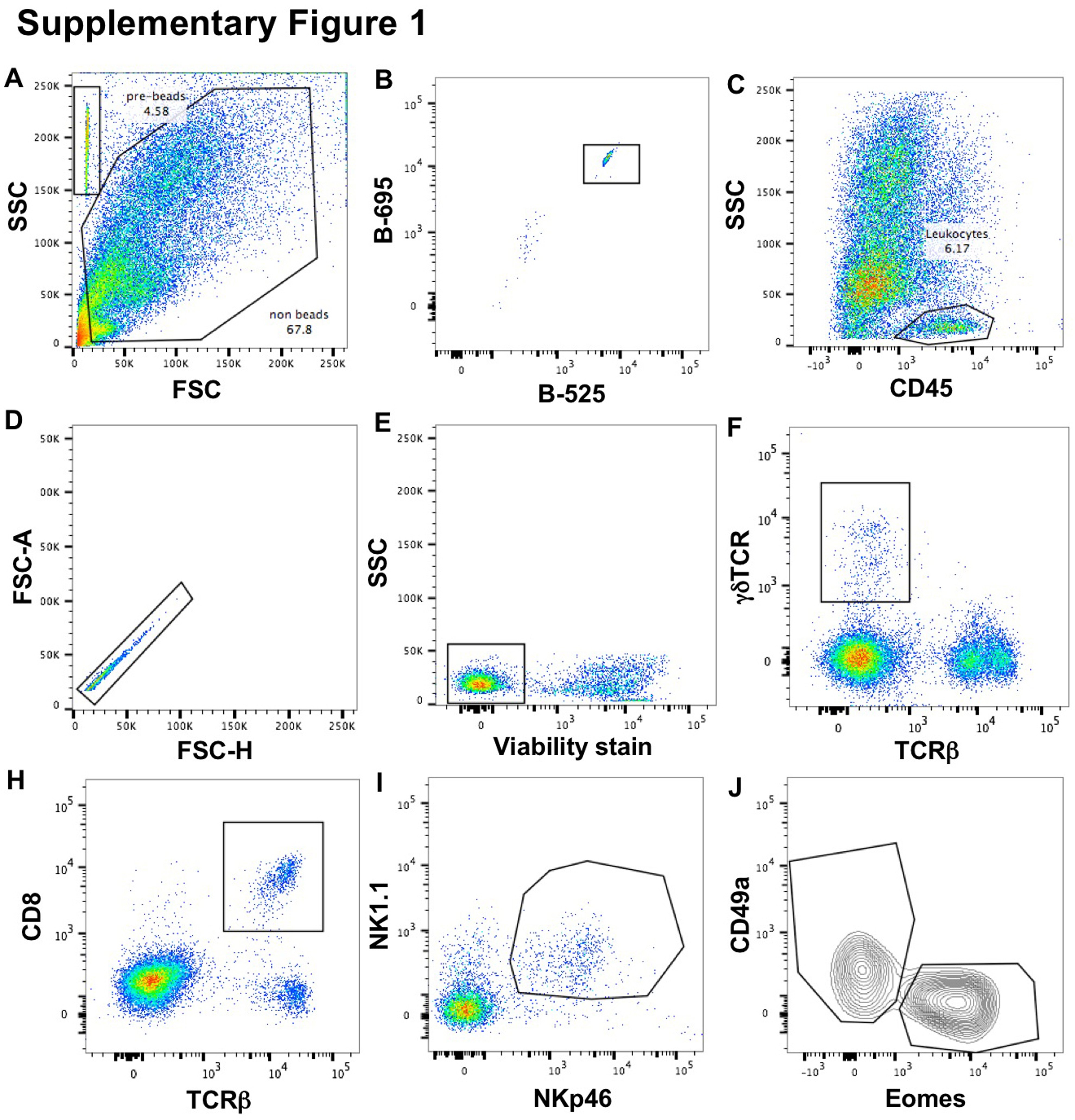

Supplement: Supplementary Figure 1 [file cddis2016215x1.tif]

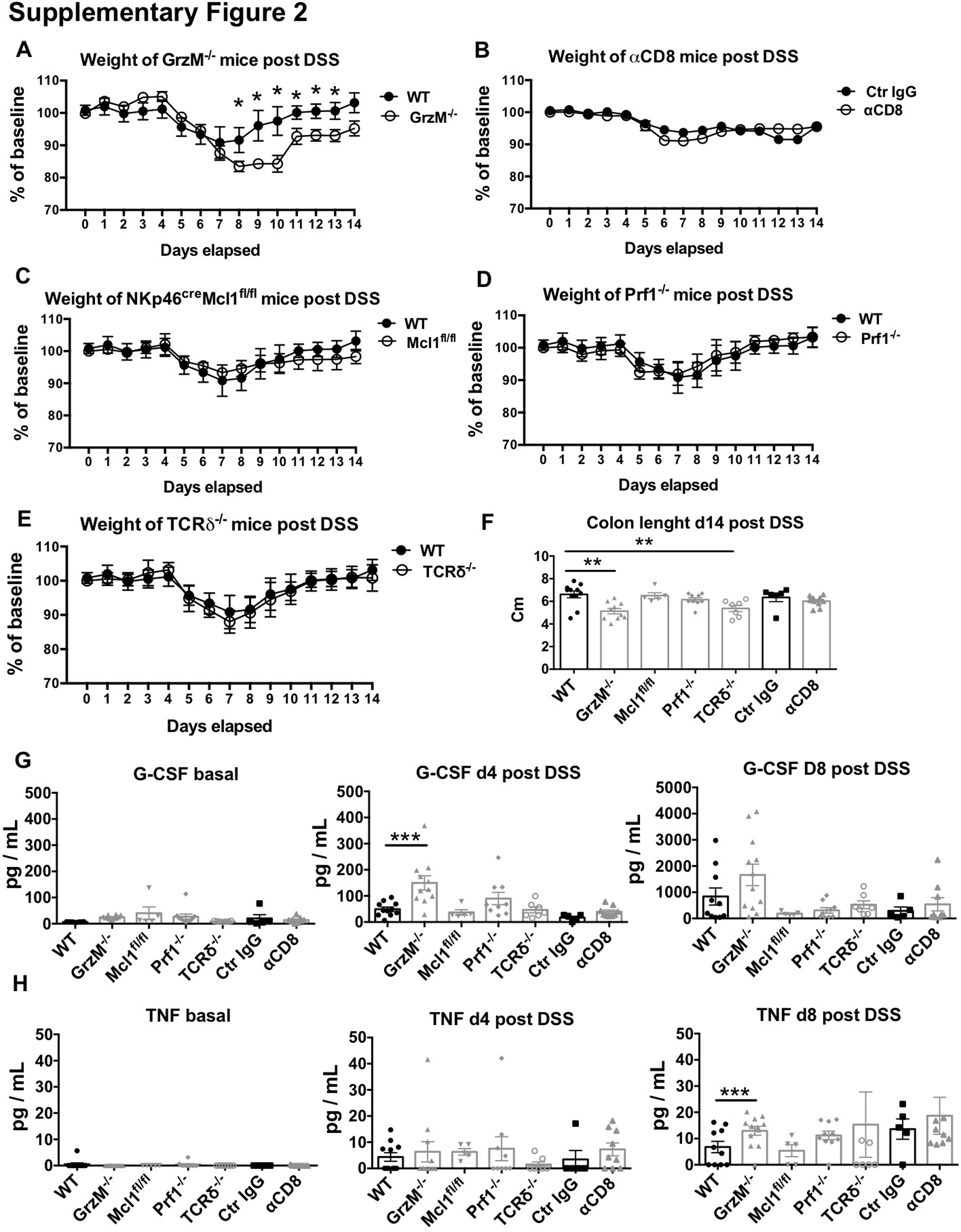

Supplement: Supplementary Figure 2 [file cddis2016215x2.tif]

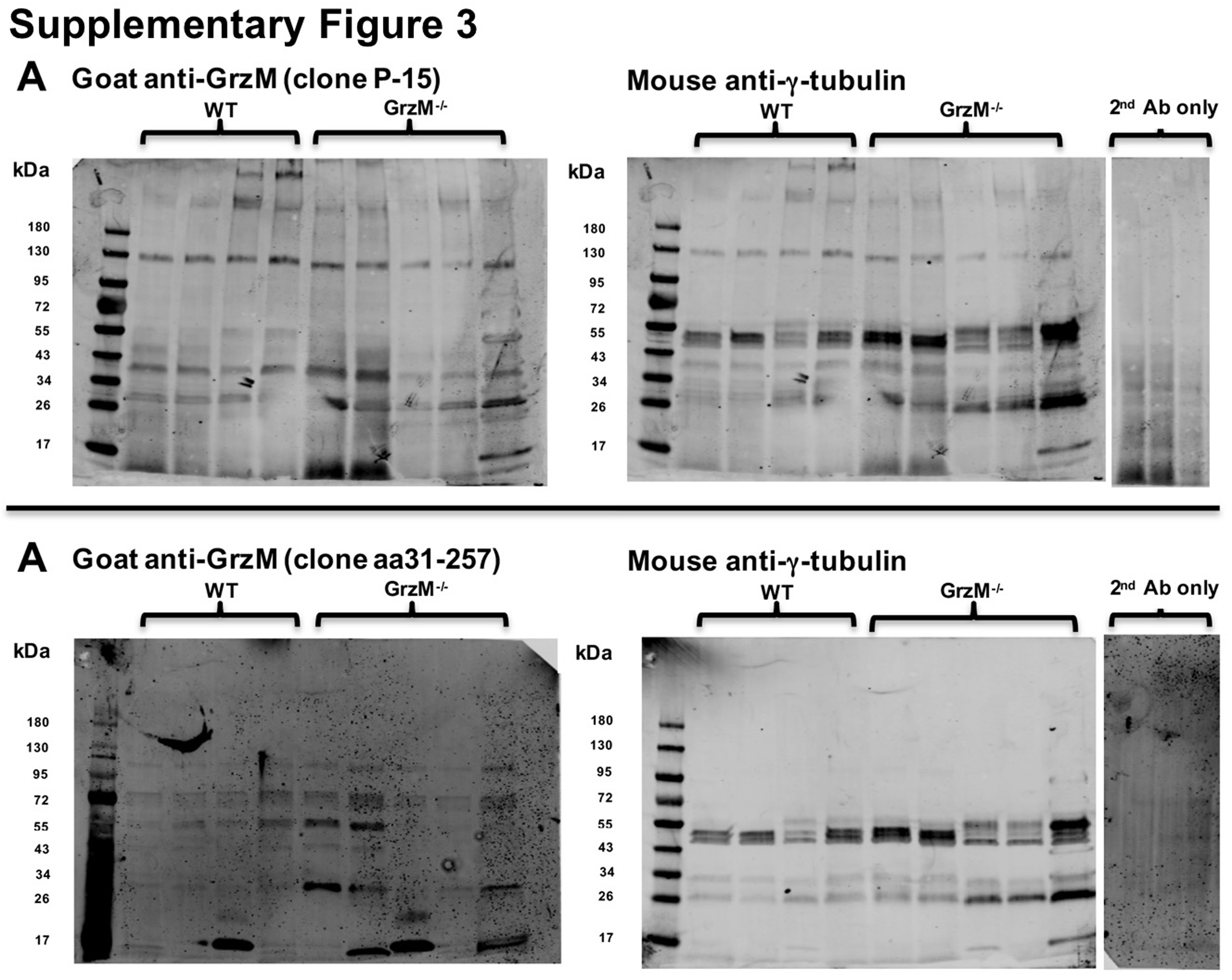

Supplement: Supplementary Figure 3 [file cddis2016215x3.tif]

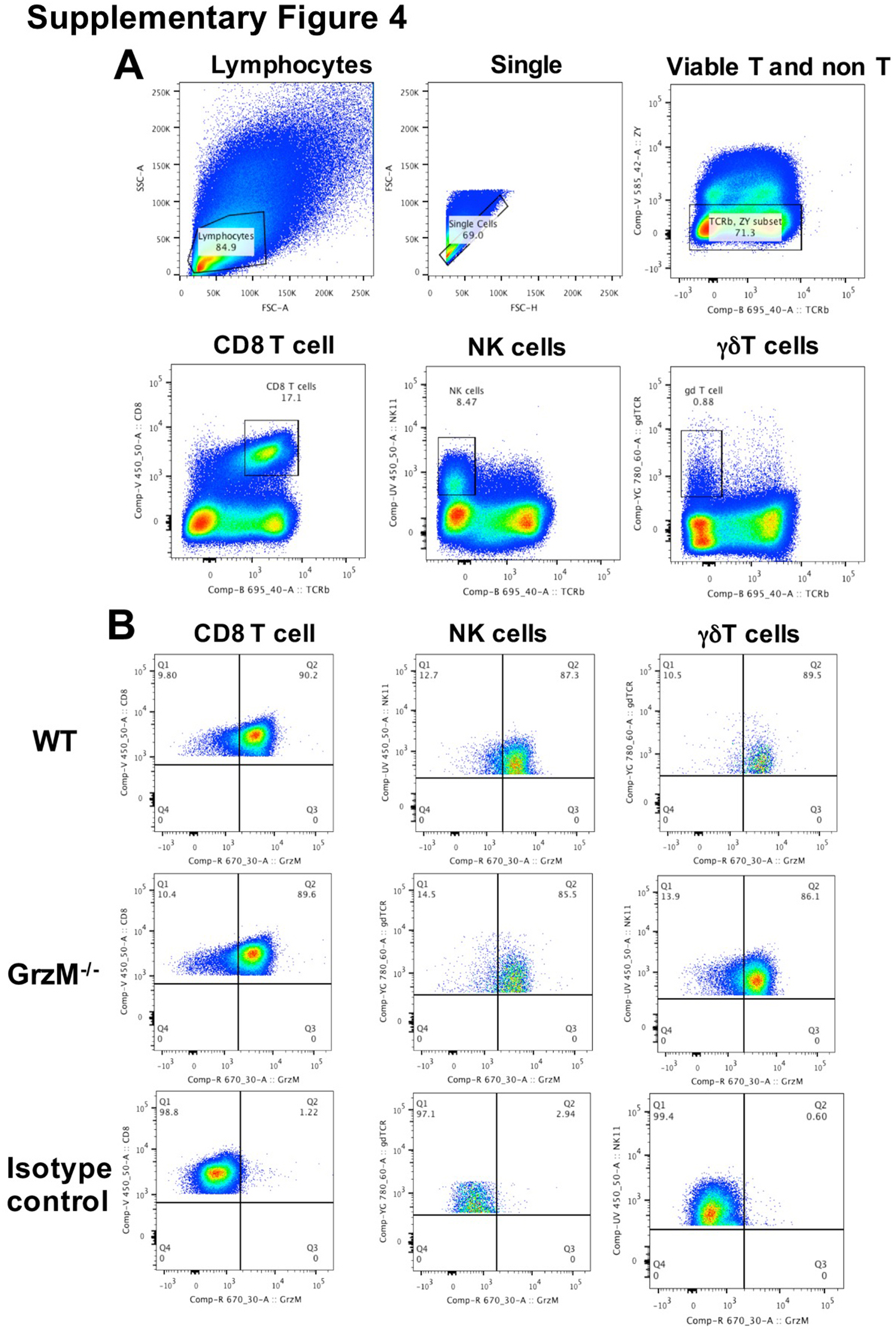

Supplement: Supplementary Figure 4 [file cddis2016215x4.tif]

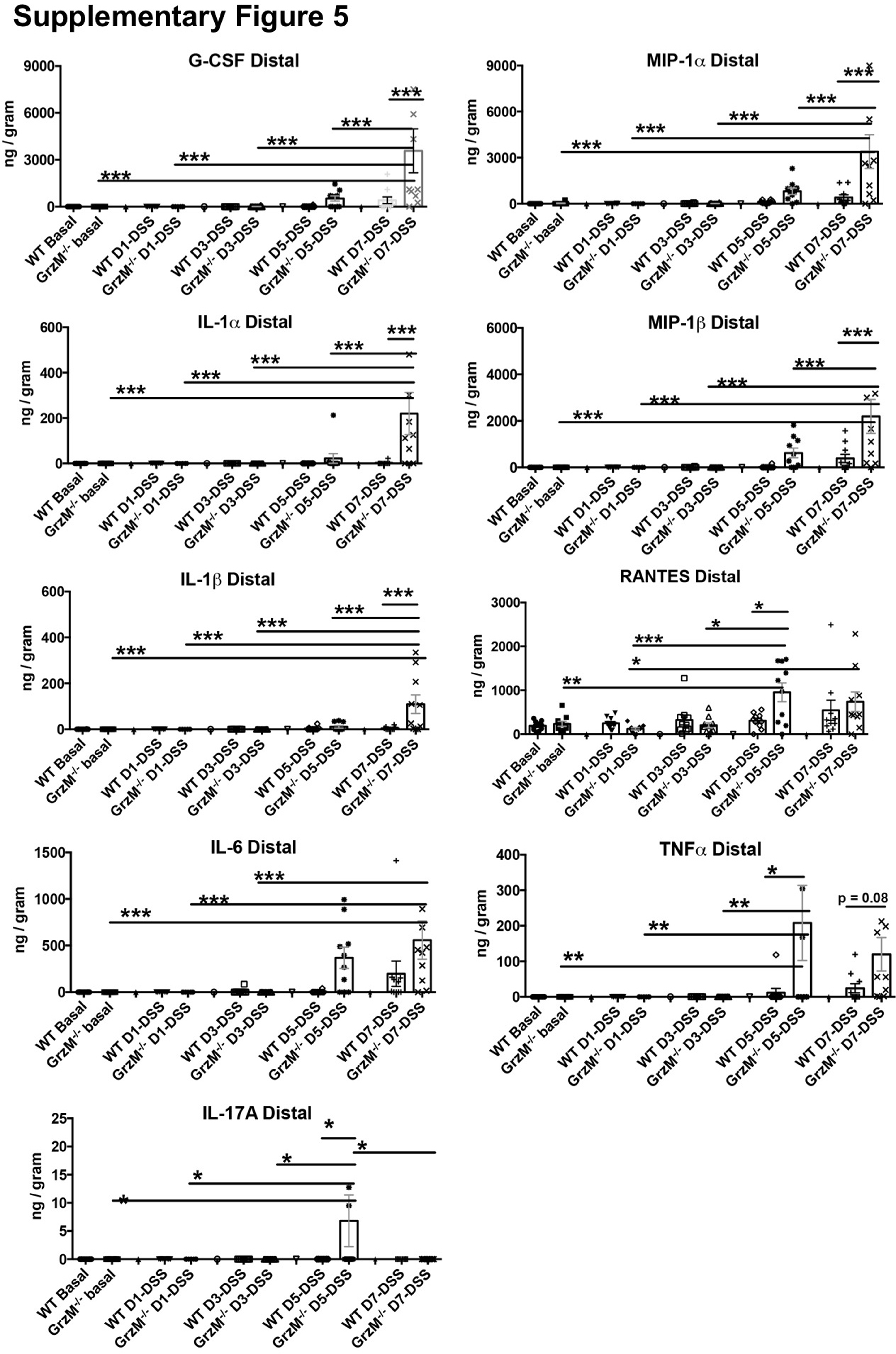

Supplement: Supplementary Figure 5 [file cddis2016215x5.tif]

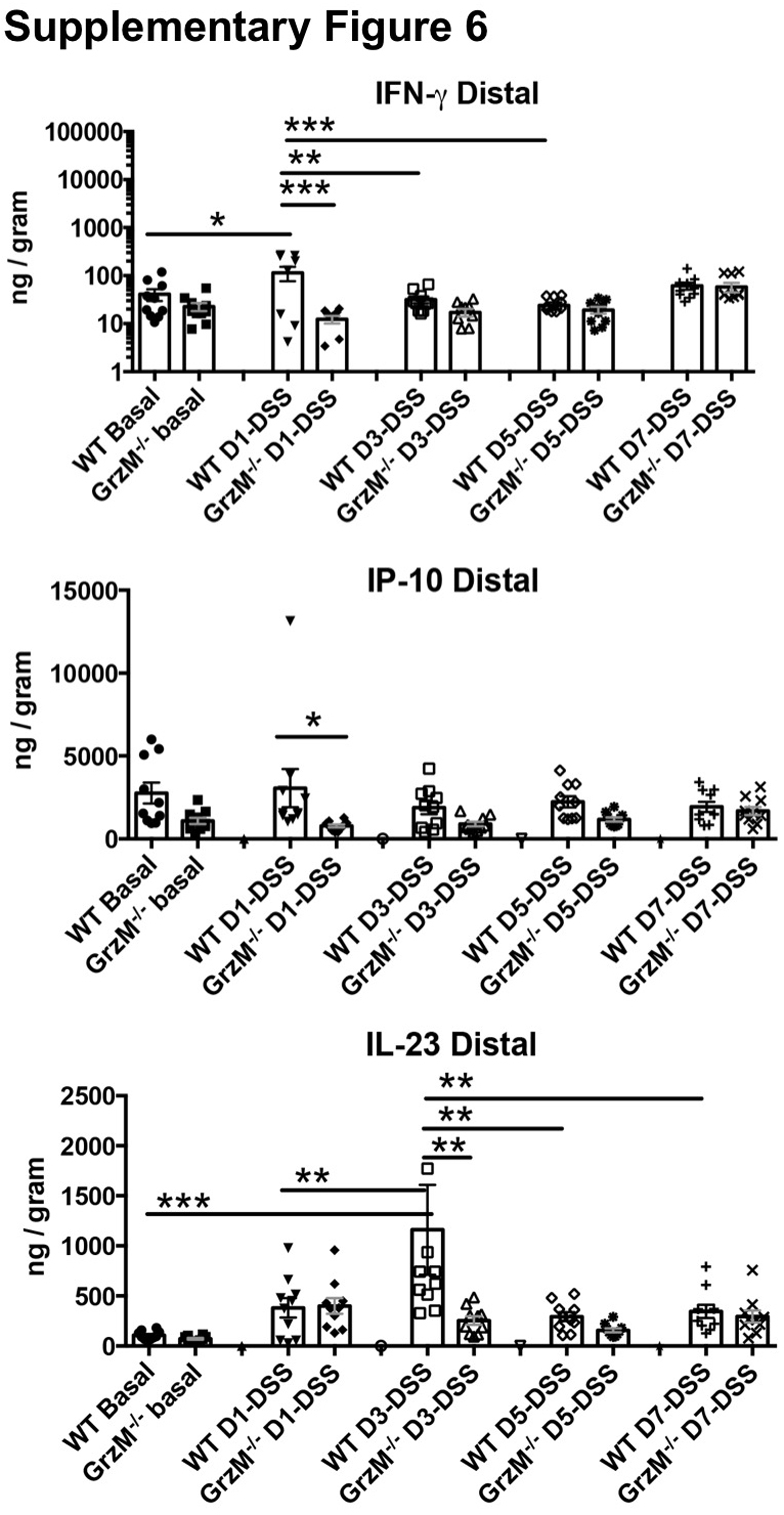

Supplement: Supplementary Figure 6 [file cddis2016215x6.tif]
