# Supplementary material for: Granzyme M has a critical role in providing innate immune protection in ulcerative colitis
Source: Cell Death Dis. 2016 Jul 21;7(7):e2302–. doi: 10.1038/cddis.2016.215 (PMC4973354; doi:10.1038/cddis.2016.215)
Supplement: Supplementary Figure Legends [file cddis2016215x7.doc]

**Supplementary Figure 1. Gating strategy to quantify CD8 T cells, γδ T cells, cNK cells and ILC1 cells from the homogenates of colon parts**. **A:** Colon homogenates were initially characterized by FSC vs SSC to gate cellular and counting bead-related events. **B:** Liquid counting beads were further gated based in their fluorescence spectra by B-695 and B-525, respectively. **C:** Cell gates (in A) were then gated in SSC vs CD45 to select leukocytes. Leukocyte events were then selected as single cells by FSC-A vs FSC-H (**D**), and viable cells (**E**). From the viable leukocyte gate, γδ T cells were gated as γδTCR^+^TCRβ^neg^ (**F**), CD8 T cells as CD8^+^TCRβ^+^ (**H**). Conventional NK (cNK) and ILC1 cells we first gated as γδTCR^neg^TCRβ^neg^NK1.1^+^NKp46^+^ (**I**), and then gated as CD49^neg^Eomes^+^ for defining cNK cells and CD49a^+^Eomes^neg^ for ILC1 (**J**). Staining and dot plots are representative from one colon homogenate.

**Supplementary Figure 2. Individual deficiency in CD8 T cells, γδ T cells, NK cells or perforin do not mimic enhanced DSS-induced UC in GrzM-deficient mice.** Mice were challenged with 5% DSS in the drinking water for 4 days, and DSS-induced weight loss was measured daily throughout the 14-day experimental period for GrzM-deficient (**A**), CD8 T cell-depleted (**B**), NK cell-deficient (NKp46^cre^Mcl1^fl/fl^ mice) (**C**), Perforin (Prf1)-deficient (**D**), TCRδ-deficient mice (**E**) and the respective control groups. Statistical analysis was performed using Mann-Whitney test. **F:** Colon length at the endpoint of the experiment is represented in cm for the indicated groups. Serum G-CSF (**G**) and TNF (**H**) cytokines were measured prior (basal) and 4 - 8 days after DSS challenge from the indicated groups. Results are representative are expressed in mean ± SEM. n = 5-11 mice per group, and *P < 0.05 was considered for statistical significance.

**Supplementary Figure 3. Commercial available antibodies to mouse GrzM detection are not specific to mouse GrzM**. **A**: The antibody clone P-15 (Santa Cruz Biotechnology) was used for western blot assessment according to the manufacturer’s instructions to detect GrzM from colon homogenates in tissue protein extraction buffer in WT or GrzM-deficient samples. Gamma-tubulin was used as loading control (40KD). **B**: The antibody clone LS-C294327/aa31-257 (LSBio) was used for western blot assessment according to the manufacturer’s instructions to detect GrzM from colon homogenates in tissue protein extraction buffer in WT or GrzM^-/-^ samples. Gamma-tubulin was used as loading control (40KD). A western blot was also performed with only secondary antibody as control. Each column corresponds to a sample of one independent mouse, n = 4 WT and n = 5 GrzM^-/-^ samples.

**Supplementary Figure 4. The anti-GrzM antibody clone LS-C294327/aa31-257 is not suitable for murine GrzM detection by Flow Cytometry. A:** Gating strategy representative from one spleen homogenate where lymphocytes were selected based in FSC vs SSC, single cells (FSC-A vs FSC-H), viable cells, then gating on CD8 T cell, NK cells and γδ T cells populations. **B**: T CD8, NK and γδ T cells from WT and GrzM^-/-^ mice were assessed for intracellular staining with the anti-GrzM antibody clone LS-C294327/aa31-257. Results are representative from one experiment, n = 3 mice per group.

**Supplementary Figure 5:** Colons of WT or GrzM^-/-^ mice were harvested at different time points after 5% DSS challenge (day 0, 1, 3, 5 and 7), and distal parts were homogenized in tissue protein extraction buffer, and analyzed for the indicated cytokines/chemokines. Pool of 2 independent experiments of n = 5 mice each (total n = 10). Statistical analysis was performed using ANOVA one way followed by Tukey’s post hoc test, where *P < 0.05, **P < 0.01, or ***P < 0.001.

**Supplementary Figure 6:** Colons of WT or GrzM-deficient mice were harvested in different time points after 5% DSS challenge (day 0, 1, 3, 5 and 7), and distal parts were homogenized in tissue protein extraction buffer, and analyzed for the indicated cytokines/chemokines. Pool of 2 independent experiments of n = 5 mice each (total n = 10). Statistical analysis was performed using ANOVA one way followed by Tukey’s post hoc test, where *P < 0.05, **P < 0.01, or ***P < 0.001.
